# A Regularized Regression Thermal Error Modeling Method for CNC Machine Tools under Different Ambient Temperatures and Spindle Speeds

**DOI:** 10.3390/s23104916

**Published:** 2023-05-19

**Authors:** Xinyuan Wei, Honghan Ye, Jinghuan Zhou, Shujing Pan, Muyun Qian

**Affiliations:** 1 School of Electrical and Information Engineering, Anhui University of Technology, Ma’anshan 230009, China; 2 Department of Industrial and Systems Engineering, University of Wisconsin—Madison, Madison, WI 53705, USA

**Keywords:** CNC machine tools, thermal error modeling, regularization, least absolute regression, practicability

## Abstract

Establishing a mathematical model to predict and compensate for the thermal error of CNC machine tools is a commonly used approach. Most existing methods, especially those based on deep learning algorithms, have complicated models that need huge amounts of training data and lack interpretability. Therefore, this paper proposes a regularized regression algorithm for thermal error modeling, which has a simple structure that can be easily implemented in practice and has good interpretability. In addition, automatic temperature-sensitive variable selection is realized. Specifically, the least absolute regression method combined with two regularization techniques is used to establish the thermal error prediction model. The prediction effects are compared with state-of-the-art algorithms, including deep-learning-based algorithms. Comparison of the results shows that the proposed method has the best prediction accuracy and robustness. Finally, compensation experiments with the established model are conducted and prove the effectiveness of the proposed modeling method.

## 1. Introduction

Thermal error is one of the main factors affecting the accuracy of CNC machine tools [1], which are gradually becoming dominant with the improvement of machine tool accuracy. In general, there are two main approaches to solving the problem of thermal errors. The first approach is to establish a simulation model based on some physical properties that can be used to simulate the thermal deformation of the structure [2]. However, this approach suffers by determining consistent boundary conditions and building an exact physical model in practice. Alternatively, the second approach is to establish a mathematical model to predict thermal errors. This approach has been widely used in practice and has been the subject of a large amount of research [1,3]. In general, a mathematical thermal error modeling method includes two key steps. The first step is to select temperature variables for modeling and is called temperature-sensitive point (TSP) selection. The temperature variables refer to the values measured by temperature sensors at different positions of the machine tool. TSP selection can simplify the model structure or mitigate the collinearity between temperature variables. The idea of TSP selection is to first classify temperature variables into different clusters and then select the most important one from each cluster [4]. This can effectively prevent the temperature variables from being strongly correlated, and the number of modeling temperature variables (MTVs) is reduced at the same time.

In the second step of a mathematical thermal error modeling method, establishing a thermal error prediction model is the key to achieving satisfactory prediction effects. Various algorithms have been applied to thermal error modeling, such as backpropagation neural network (NN) [5], support vector machine [6], multiple linear regression [7,8], state-space [9], and Gaussian process regression (GPR) [10]. Notably, the NN [5] only consists of a single hidden layer whereas the deep learning method is more complicated with multiple hidden layers. In addition, ridge regression [11] and principal component regression [12] algorithms have been used to solve the collinearity between temperature variables. Recently, deep learning algorithms have been adopted for thermal error modeling to further improve prediction accuracy. For example, Fujishima et al. [13] proposed a novel deep-learning thermal error compensation method in which the compensation weight can be changed adaptively according to the reliability of thermal displacement prediction. The deep learning convolutional NN algorithm [14], bidirectional long short-term memory (LSTM) deep learning algorithm [15], and stacked LSTM algorithm [16] have all been used for thermal error modeling. Furthermore, several researchers have built hybrid thermal error models by combining different algorithms to take advantage of their respective features [17,18,19,20,21]. More recently, digital twin technology was adopted by [22] to solve the problem of thermal errors. The authors utilized the digital twin concept to propose a self-learning-empowered error control framework for the real-time thermal error prediction and control.

The above studies provide various solutions to thermal errors. However, thermal error models, especially for those based on the deep learning method, have several limitations, including a very complex structure, requiring a large amount of training data, and a lack of interpretability. As a result, these methods are difficult to deploy in practical engineering for thermal error compensation of machine tools. In other words, in addition to prediction accuracy, robustness [23], and adaptability [24], practicality should be considered as another important indicator to effectively solve the engineering problem of thermal error modeling and compensation. From this perspective, it is suggested that traditional regression algorithms are more suitable. While traditional regression algorithms have a simple model structure and good interpretability, the prediction effects of the regression algorithms in the existing literature are not as good as those of deep learning algorithms. Therefore, there is a research gap: the existing literature lacks a thermal error modeling method that has a simple structure, good interpretability, and comparable performance of prediction effects to deep learning algorithms.

To fill the research gap, a new method based on a regularized regression algorithm is proposed to enhance the prediction ability of the regression algorithm. In particular, the least absolute regression algorithm is first used for thermal error modeling. To improve the robustness of the established model, both L1 and L2 regularizations are used by shrinking the regression coefficients. Accordingly, the stability of the regression model is improved. In addition, the proposed modeling method can automatically select TSPs owing to the presence of L1 regularization. Further, multiple batches of experimental data are used for modeling to ensure the sufficiency of thermal error information, which is a key prerequisite for effective modeling. Through analysis, the optimal combination of the number of MTVs and the coefficients of different regularization terms can be obtained, which is further used for thermal error modeling by the regularized regression algorithm. In summary, there are two main contributions of the proposed thermal error modeling method: (1) the least absolute regression algorithm, integrated with L1 and L2 regularization, is able to automatically select TSPs and reduce the collinearity simultaneously, thereby enhancing the prediction ability; (2) the prediction effects of the proposed thermal error modeling method are better with the simple model structure, compared with the state-of-the-art algorithms, including complex deep-learning-based algorithms.

Section 2 introduces the thermal error measurement experiments. Afterward, the existing thermal error modeling algorithms are briefly described in Section 3, including TSP selection and modeling algorithms. The proposed thermal error modeling method based on a regularized regression algorithm is introduced in Section 4. The effects of the number of MTVs and the coefficients of regularization terms are systematically analyzed for the proposed modeling method in Section 5. In Section 6, the proposed modeling method is compared with the state-of-the-art algorithms and verified by actual compensation experiments. Finally, conclusions are drawn in Section 7.

## 2. Thermal Error Modeling Algorithms

To obtain sufficient experimental data, 46 batches of thermal error experiments were conducted within a year. The environment temperatures and spindle speeds of these experiments are different.

### 2.1. Experimental Object

**Figure 1:**
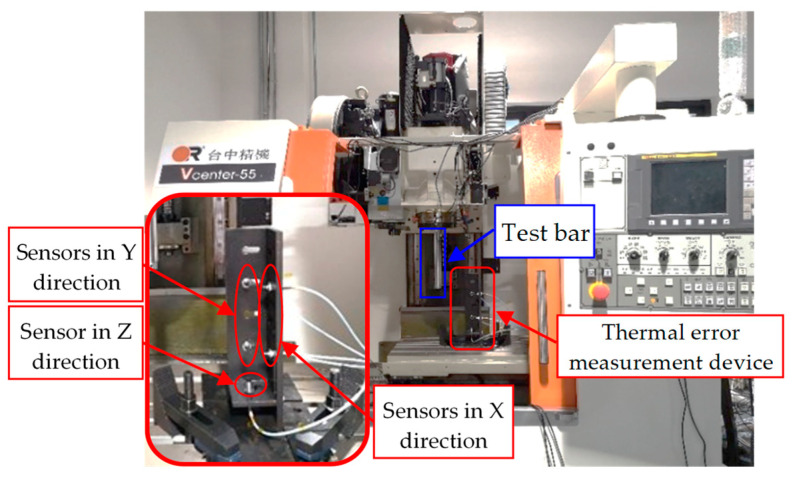
Experiment object.

In this study, a vertical three-axis machining center (Vcenter-55 type) is regarded as the experimental object, as shown in Figure 1. The thermal errors of the spindle are measured using five displacement sensors, which are capacitive with a measurement accuracy of 1 μm. The thermal error can be obtained by subtracting the initial measurement value from each displacement measurement according to ISO 230-3: 2020 [25].

**Figure 2:**
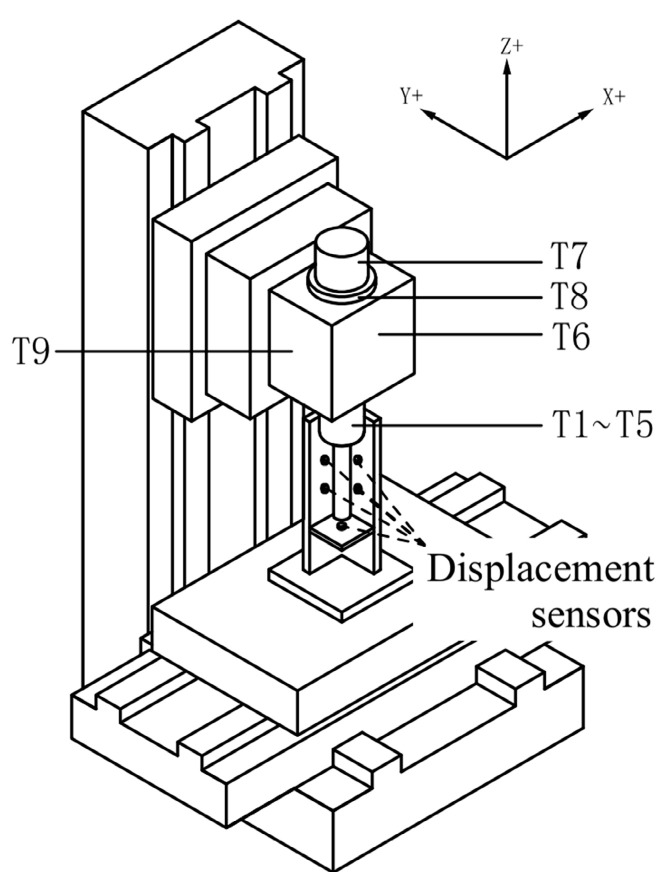
Positions of temperature sensors.

Ten platinum resistance temperature sensors (measurement accuracy: 0.1 °C) are arranged at different key positions of the machining center. Table 1 shows the distribution and function of these 10 sensors. The positions of these sensors are shown in Figure 2. Note that T10 is not displayed in Figure 2 since it was installed on the machine body frame to measure the ambient temperature.

### 2.2. Experimental Arrangements

The experiments were conducted according to ISO 230-3: 2020 [25]. The 46 batches of experiments were sorted based on initial environment temperature and were labeled K1–K46. The experimental parameters are shown in Table 2. The spindle speeds of these experiments were set to three grades: 2000 revolutions per minute (rpm), 4000 rpm, and 6000 rpm. Each batch of the experiment lasted at least 6 h. In each batch of experiments, the spindle idled and the worktable ran back and forth at a constant feed rate. The experimental data were recorded every 5 min, including thermal error and temperature.

### 2.3. Experimental Data Analysis

**Figure 3:**
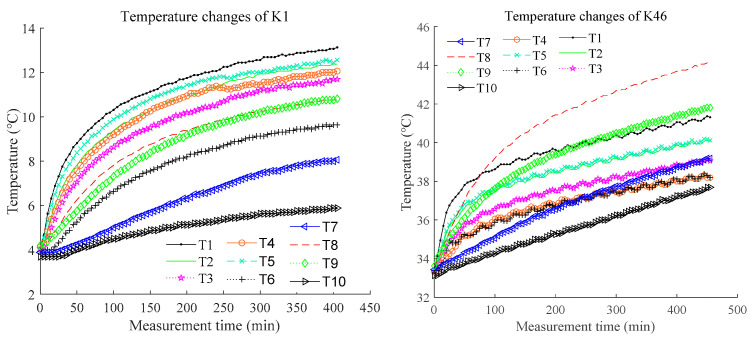
Temperature changes corresponding to K1 and K46.

Since the experiments in this study were conducted throughout an entire year, the range of the initial environmental temperature for K1–K46 is large, namely 3.7–32.2 °C. The temperature changes of K1 and K46 are shown in Figure 3, which illustrates the differences in temperature changes of the machining center under different ambient temperatures.

The environment temperature of the 46 batches of experiments can be divided into three different intervals: below 13.2 °C, 13.2–22.7 °C, and over 22.7 °C. The values of 13.2 °C and 22.7 °C are the trisection points of the initial temperature range 3.7–32.2 °C. Combined with three ambient temperature intervals and three spindle speeds, nine different experimental conditions can be divided, as shown in Table 3.

**Figure 4:**
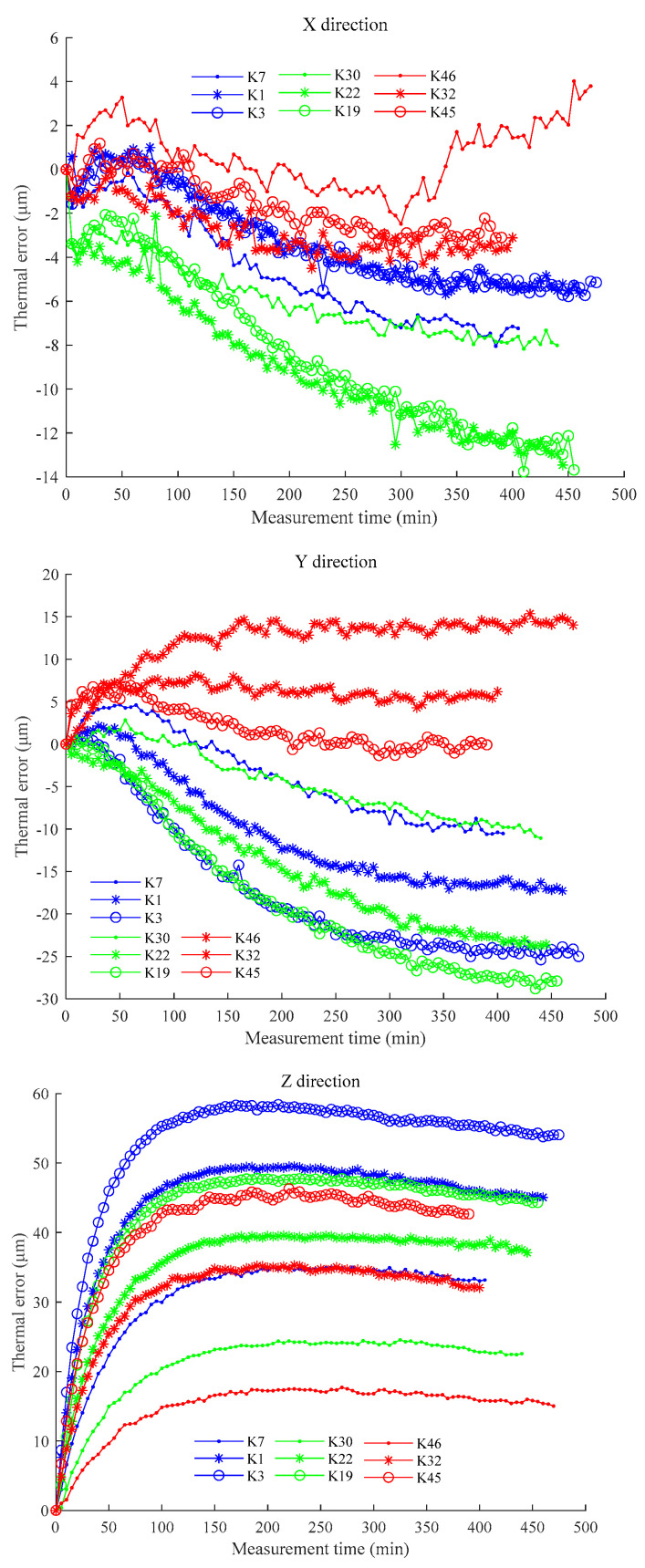
Thermal error curves of the selected nine batches of experiments.

One batch of experimental data can be randomly selected from each experimental condition, and a total of nine batches of data can be selected for analysis. In these nine batches of experiments, every three batches of experiments are in the same ambient temperature range, and the spindle speeds are 2000, 4000, and 6000 rpm, respectively. For example, K7, K1, K3, K30, K22, K19, K46, K32, and K45 are selected. The thermal errors of the selected nine batches of experiments are shown in Figure 4 to show the differences in thermal errors under different experimental conditions.

Figure 4 shows that the thermal errors are significantly different under different experimental conditions. For example, the lower the ambient temperature, the larger the thermal error in the Z direction under the same spindle speed. Within the same ambient temperature interval, the higher the spindle speed, the larger the thermal error. That is to say, the thermal error is affected by both the spindle speed and ambient temperature change. For instance, the thermal error of K5 is the largest among the nine batches of experiments due to the lowest ambient temperature and the highest spindle speed. On the contrary, the thermal error of K44 is the smallest because of the highest ambient temperature and the lowest spindle speed. The phenomenon above indicates that the thermal errors contain different information under different experimental conditions. Note that for each experiment, all temperature measurements are subtracted from the initial ambient temperatures for thermal error modeling.

## 3. Existing Thermal Error Modeling Algorithms

In this section, the existing TSP selection and modeling algorithms are introduced, respectively. Specifically, Section 3.1 introduces the correlation coefficient TSP selection algorithm. Section 3.2 introduces the ARX, LSTM, and GPR thermal error modeling algorithms.

### 3.1. TSP Selection Algorithm Based on Correlation Efficient

The rth temperature variable is $x_{r}={\left[x_{r1},x_{r2},\ldots ,x_{rn}\right]}^{T}$, where n is the number of data samples. The temperature data can be recorded as $X=\left(x_{1},x_{2},\ldots x_{r}\ldots x_{p}\right)$, where p is the number of temperature variables. The thermal error is $y={\left[y_{1},y_{2},\ldots ,y_{n}\right]}^{T}$. Then the correlation coefficient $\rho_{x_{r}y}$ can be calculated as

(1) $$\rho_{x_{r}y}=\frac{Cov\left(x_{r},y\right)}{\sqrt{Var\left(x_{r}\right)Var\left(y\right)}},$$

where $Var\left(\cdot \right)$ represents the variances of the corresponding variable and $Cov\left(x_{r},y\right)$ is the covariance between $x_{r}$ and y. Specifically,

(2) $$Cov\left(x_{r},y\right)=\frac{\sum_{i=1}^{n}\left(x_{r,i}-{\bar{x}}_{r}\right)\left(y_{i}-\bar{y}\right)}{n-1},$$

(3) $$Var\left(x_{r}\right)=\frac{\sum_{i=1}^{n}{\left(x_{ri}-{\bar{x}}_{r}\right)}^{2}}{n-1},$$

(4) $$Var\left(y\right)=\frac{\sum_{i=1}^{n}{\left(y_{i}-\bar{y}\right)}^{2}}{n-1}.$$

where ${\bar{x}}_{r}$ and $\bar{y}$ are the average values of $x_{r}$ and y, respectively. This correlation coefficient method selects the temperature variables that are highly correlated with the thermal error as TSPs. However, this method may lose important environmental temperature information, which has significant effects on thermal errors [4] because the environmental temperature variable is rarely selected as a TSP.

### 3.2. Thermal Error Modeling Methods Based on ARX, LSTM, and GPR Algorithms

In this subsection, three state-of-the-art algorithms are briefly introduced, the ARX, LSTM, and GPR algorithms. The ARX thermal error model can be expressed as shown below.

(5) $$y_{t}=\alpha_{0}+\overset{m_{a}}{\underset{i=1}{\sum }}\alpha_{i}y_{t-i}+\overset{p}{\underset{j=1}{\sum }}\overset{m_{b}}{\underset{i=1}{\sum }}\beta_{j,i}x_{j,t-i},$$

where $y_{t-i}=$ $x_{j,t-i}=0$ when t≤i for j=1,…p, and t varies from 1 to n. In addition, $m_{a}$ is the order of auto-regression and $m_{b}$ is the length of the input memory. The output $y_{t}$ is influenced by its past $m_{a}$ values and the past $m_{b}$ values of the input. Finally, $\alpha_{i}$ and $\beta_{j,i}$ are the coefficients of $y_{t-i}$ and $x_{j,t-i}$, respectively.

There are many studies establishing thermal error prediction models using the LSTM neural network algorithm [8,26,27]. In this study, the LSTM neural network is adopted as another baseline method and is available in MATLAB software. Specifically, the LSTM method contains a sequence input layer and a regression output layer. At the same time, an LSTM layer and a fully connected layer are designed. For more details on the structure of the LSTM and its training options, please refer to [10].

The GPR algorithm can be used for thermal error modeling [10], which has good prediction effects. The GPR algorithm is selected as the third baseline method for prediction comparison in this study. It is noteworthy that although the GPR thermal error modeling algorithm can automatically select the TSPs, the TSP selection is conducted in an iterative procedure. However, our proposed method automatically selects TSPs in a single step, which is more straightforward and computationally inexpensive.

## 4. The Proposed Thermal Error Modeling Method

As has been noted, the existing thermal error modeling algorithms lack simple structure, good interpretability, and comparable performance of prediction effects to deep learning algorithms. To fill this gap, we propose a new method based on the regularized regression model in this section. Specifically, the regularized regression model is first formulated in Section 4.1. Then, the solution to the regularized regression model is provided in Section 4.2.

### 4.1. Thermal Error Modeling Based on Regularized Regression

The multiple linear regression thermal error y model concerning temperature variables $x_{1},x_{2},\ldots x_{r}\ldots x_{p}$ can be expressed as

(6) $$y=\beta_{0}+\beta_{1}x_{1}+\beta_{2}x_{2}+\ldots +\beta_{p}x_{p}+\varepsilon ,$$

where $\beta ={\left[\beta_{0},\beta_{1},\beta_{2},\ldots ,\beta_{p}\right]}^{T}$ represent the coefficients of the model. ε is the random error obeying N(0, $\sigma^{2}$), where $\sigma^{2}$ is the variance of the normal distribution.

According to the least-squares algorithm, β can be calculated by minimizing the objective function as shown below.

(7) $$\min Q_{1}\left(\beta \right)=\min{\left(y-A\beta \right)}^{T}\left(y-A\beta \right),$$

where $A=\left[I,X\right]$ and I indicate an n-dimensional unit column vector.

Then the regression coefficients can be calculated in the closed-form equation as

(8) $$\hat{\beta }={\left[{\hat{\beta }}_{0},{\hat{\beta }}_{1},{\hat{\beta }}_{2},\ldots ,{\hat{\beta }}_{p}\right]}^{T}={\left(A^{T}A\right)}^{-1}A^{T}y,$$

As pointed out in the existing literature [11], the least-squares algorithm is sensitive to outliers and collinearity between independent variables. The collinearity would lead to $\left|A^{T}A\right|\approx 0$ and then the values of the main diagonal elements of $A^{T}A^{-1}$ are large. As a result, the variance of the estimated regression coefficients $\hat{\beta }$ is large, as shown below.

(9) $$Var\left(\hat{\beta }\right)=Cov\left(\hat{\beta },\hat{\beta }\right)=\sigma^{2}A^{T}A^{-1}.$$

To solve this problem, the ridge regression algorithm replaces the matrix $A^{T}A$ in Equation (4) with $A^{T}A+\lambda_{2}E$. Then the coefficients can be estimated as

(10) $$\hat{\beta }={\left(A^{T}A+\lambda_{2}E\right)}^{-1}A^{T}y,$$

where E represents an n-dimensional identity matrix. $\lambda_{2}$ is called the ridge parameter.

From the optimization point of view, the object function of the ridge regression algorithm adds the L2 regularization term as a penalty term based on the least-squares algorithm, as shown below.

(11) $$\min Q_{2}\left(\beta \right)=\min\left[{\left(y-A\beta \right)}^{T}\left(y-A\beta \right)+\lambda_{2}\overset{p}{\underset{j=1}{\sum }}\beta_{j}{}^{2}\right].$$

Further, the least absolute shrinkage and selection operator (LASSO) algorithm takes the L1 regularization term as the penalty term to select important variables involved in the model. Then the objective function is

(12) $$\min Q_{3}\left(\beta \right)=\min\left[{\left(y-A\beta \right)}^{T}\left(y-A\beta \right)+\lambda_{1}\overset{p}{\underset{j=1}{\sum }}\left|\beta_{j}\right|\right].$$

In addition, the elastic-net regression (ENR) algorithm combines the L1 and L2 regularization to construct the penalty terms. The object function of the ENR algorithm is

(13) $$\min Q_{4}\left(\beta \right)=\min\left[{\left(y-A\beta \right)}^{T}\left(y-A\beta \right)+\lambda_{1}\overset{p}{\underset{j=1}{\sum }}\left|\beta_{j}\right|+\lambda_{2}\overset{p}{\underset{j=1}{\sum }}\beta_{j}{}^{2}\right],$$

where $\lambda_{1}$ and $\lambda_{2}$ are the coefficients of the L1 and L2 regularization terms, respectively.

It can be found from Equation (13) that the ENR algorithm includes the least-squares regression, ridge regression, and LASSO. When $\lambda_{1}=\lambda_{2}=0$, Equation (13) is the objective function of the least-squares algorithm. When $\lambda_{1}=0$ and $\lambda_{2}\neq 0$, Equation (13) is the objective function of the ridge regression algorithm. When $\lambda_{1}\neq 0$ and $\lambda_{2}=0$, Equation (13) is the objective function of the LASSO algorithm.

Compared with the least-squares regression, the least-absolute regression has better robustness. The object function of the least-absolute algorithm is shown below.

(14) $$\min Q_{5}\left(\beta \right)=\min\left|y-A\beta \right|.$$

Similarly, the L1 and L2 regularization can also be applied to Equation (14), then the objective function of Equation (14) can be updated as follows.

(15) $$\min Q_{6}\left(\beta \right)=\min\left[\left|y-A\beta \right|+\lambda_{3}\overset{p}{\underset{j=1}{\sum }}\left|\beta_{j}\right|+\lambda_{4}\overset{p}{\underset{j=1}{\sum }}\beta_{j}{}^{2}\right],$$

where $\lambda_{3}$ and $\lambda_{4}$ are the coefficients of the L1 and L2 regularization terms, respectively.

The regression coefficients can be estimated by solving Equation (15), which is called the least absolute elastic-net regression (LAENR) algorithm in this study. As a result, the LAENR algorithm can not only select important variables like LASSO regression but also inherits the stability of ridge regression. Furthermore, with the use of the least-absolute algorithm, the LAENR algorithm has better robustness.

**Figure 5:**
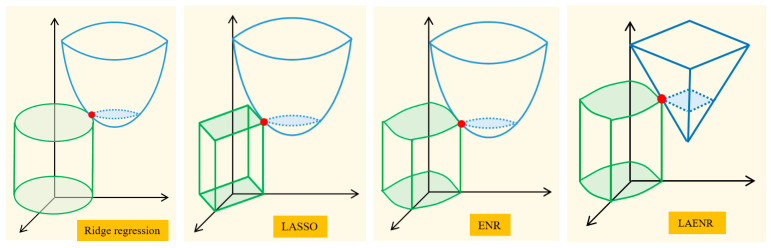
Schematic diagram of optimal solutions of the ridge regression, LASSO, ENR, and LAENR algorithms.

To intuitively show the differences between the ridge regression, LASSO, ENR, and LAENR algorithms, the case of only two independent variables is taken as an example to illustrate the optimal solutions to Equations (11)–(13) and (15) (Figure 5). In Figure 5, the blue parts represent the original objective function of Equation (7). The green parts represent the regularization terms, which indicates that the regularization terms limit the value of the model coefficients. It can be observed that the optimal solutions of LASSO, ENR, and LAENR algorithms can easily fall on a coordinate axis. In this case, the coefficient of the independent variable on the other coordinate axis is zero. As a result, the variable selection is realized. The reason for this situation is the existence of L1 regularization, which restricts the feasible region of the optimal solution to a region with cusps. As a comparison, the ridge regression algorithm cannot select variables since the coefficients are close to zero but not equal to zero. The shape of the objective function changes from a paraboloid (LASSO and ENR) to a conical surface (LAENR), which demonstrates the difference between the least-squares and the least-absolute algorithms.

### 4.2. Solution to the Least-Absolute Regularized Regression

Solving Equation (15) is an unconstrained nonlinear multivariable minima problem. Since there is an operation to solve the absolute value of the objective function, there is no continuous first derivative. As a result, the analytical solution to this problem does not exist. Therefore, the quasi-Newton method [28] is adopted to obtain the optimal solution quickly and reliably. In the Newton method, the minimum $\;\beta^{*}$ of objective function $Q_{6}\left(\beta \right)$ can be calculated by the iterative equation as shown below.

(16) $$\beta_{k+1}=\beta_{k}-H_{k}{}^{-1}g_{k},$$

where $H_{k}=H\left(\beta_{k}\right)={\left[\frac{\partial Q_{6}}{\partial \beta_{i}\beta_{j}}\right]}_{p\times p}$, which is called the Hessian matrix, and k is the index of the kth iteration. $g_{k}=g\left(\beta_{k}\right)=\nabla Q_{6}\left(\beta_{k}\right)$, which represents the value of the gradient vector of $Q_{6}$ at point $\beta_{k}$.

The difference between the quasi-Newton and Newton methods is to solve the inverse of the Hessian matrix. In the quasi-Newton method, the inverse of the Hessian matrix is represented by an approximate positive definite symmetric matrix, thus avoiding the calculation of second-order partial derivatives. There are other methods to construct the inverse of the Hessian matrix, such as the Davidon–Fletcher–Powell method [29]. In this study, the inverse of the Hessian matrix is constructed using the Broyden–Fletcher–Goldfarb–Shanno method, which is generally considered to be the most efficient. The iterative equation of the Hessian matrix is shown below.

(17) $$H_{k+1}=H_{k}+\frac{y_{k}y_{k}{}^{T}}{y_{k}{}^{T}\delta_{k}}-\frac{H_{k}\delta_{k}\delta_{k}{}^{T}H_{k}}{\delta_{k}{}^{T}H_{k}\delta_{k}},$$

where $y_{k}=Q_{6}\left(\beta_{k}\right)$, $\delta_{k}=\beta_{k+1}-\beta_{k}$.

Based on the above iterative calculation, the optimal solution $\beta^{*}$ to the problem of Equation (15) can be obtained. To minimize the influence of the local minimum problem and obtain the global optimal solution, a multi-start algorithm is adopted. The multi-start algorithm first generates many start points within the feasible region. Then the quasi-Newton method is used to solve Equation (15) at each start point. Finally, the global optimal solution is obtained from all the solutions corresponding to the start points.

Note that the temperature and thermal error data are normalized before modeling. The normalized method is shown below.

(18) $$\left\{\begin{array}{c} x_{r}{}^{*}=\frac{x_{r}-\bar{x_{r}}}{\sigma_{x_{r}}}\\ y^{*}=\frac{y-\bar{y}}{\sigma_{y}} \end{array}\right.,$$

where $\bar{x_{r}}$ and $\bar{y}$ represent the mean of the $x_{r}$ and y, respectively. The $\sigma_{x_{r}}$ and $\sigma_{y}$ are the standard deviations of $x_{r}$ and y, respectively.

Then the thermal error prediction model after normalization can be established based on the above modeling method, as shown below.

(19) $$y^{*}=b_{1}x_{1}{}^{*}+b_{2}x_{2}{}^{*}+\ldots +b_{p}x_{p}{}^{*}+\varepsilon ,$$

where $b_{i}\left(i=1,\;2,\;\ldots \;p\right)$ are the coefficients of the model.

The model coefficients of the original data β can be obtained by the follow formula:

(20) $$\left\{\begin{array}{c} \beta_{i}=b_{i}*\frac{\sigma_{y}}{\sigma_{y}}\\ \beta_{0}=\bar{y}-\overset{p}{\underset{i=1}{\sum }}b_{i}*\bar{x_{i}} \end{array}\right..$$

## 5. Analysis of the Proposed Modeling Method

In this section, the Z-direction thermal error is taken as an example to show the analysis process. The number of MTVs and regularization coefficients of the proposed modeling method are analyzed respectively.

### 5.1. Analysis of Modeling Effects with Different Numbers of Temperature Variables

Although the proposed modeling method has the ability of variable selection, the number of temperature variables initially involved in the modeling has an important impact on the modeling results. To study the influence of different numbers of temperature variables for modeling, the selected nine batches of experiments in Section 2.3 are used together for thermal error modeling different numbers of temperature variables. The modeling effects with different numbers of temperature variables are analyzed.

According to the TSPs selection method in Section 3.1, the correlation coefficient between thermal error and each temperature variable can be calculated. Accordingly, the order of the temperature variables can be obtained as 1, 5, 2, 4, 3, 6, 9, 8, 7, and 10. Then the MTVs can be determined according to this order. Then the thermal error models are built with the selected nine batches of experiments by the LAENR algorithm. In addition, the regularization coefficients are set as $\lambda_{3}=1$ and $\lambda_{4}=1$ for illustration. Before modeling, the temperature data $X=\left(x_{1},x_{2},\ldots x_{r}\ldots x_{p}\right)$ are subtracted from the initial value to obtain the temperature increments $\Delta X=\left(\Delta x_{1},\Delta x_{2},\ldots \Delta x_{r}\ldots \Delta x_{p}\right)$ for modeling. Therefore, the regression model of thermal error is

(21) $$\hat{y}=\beta_{0}+\beta_{1}\Delta x_{1}+\beta_{2}\Delta x_{2}+\ldots +\beta_{p}\Delta x_{p},$$

where $\hat{y}$ represents the predicted thermal errors.

The optimal solution of the LAENR algorithm is calculated using MATLAB software. Then the modeling results, including regression model coefficients and fitting accuracy, are calculated as shown in Table 4. The fitting accuracy is characterized by the root mean square (RMS) [30] when the established model makes predictions on the modeled data itself.

**Figure 6:**
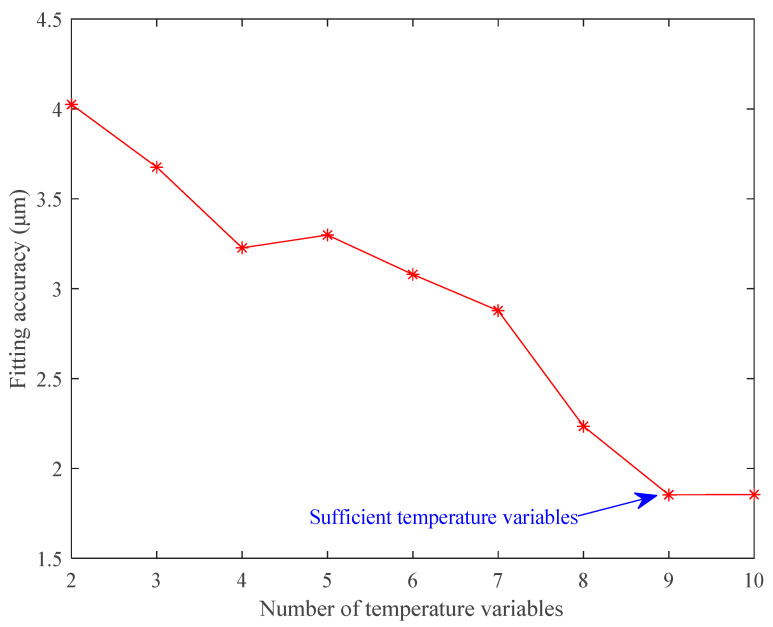
Fitting accuracy with different numbers of temperature variables.

From Table 4, we have the following observations. First, the coefficients of some temperature variables are equal to zero when the number of temperature variables is more than six. This proves that the LAENR algorithm can automatically eliminate unimportant temperature variables in the modeling process because this algorithm enjoys the sparsity characteristics of the L1 regularization. Second, the trend of fitting accuracy with the number of temperature variables, which is also shown in Figure 6, is that the fitting accuracy improves as the number of temperature variables increases. This indicates that more temperature variables involved in thermal error modeling can provide more temperature information, and thus improve the fitting accuracy. In addition, the curve becomes flat when more than nine temperature variables are included for modeling. This shows that nine temperature variables are sufficient to model the thermal error. Note that the LAENR algorithm would automatically eliminate unimportant or duplicate temperature variables from the initial modeling temperature variables.

### 5.2. Analysis of the Optimal Number of Temperature Variables and Regularization Coefficients

Based on the above analysis, it is obvious that the number of MTVs affect the thermal error prediction effects. However, there is no uniform principle for determining the number of MTVs. In addition, the values of the regularization coefficients have important influences on the thermal error prediction. Therefore, considering different numbers of MTVs and different regularization coefficients, the thermal error model is established by the selected nine batches of experimental data.

The remaining 37 batches of experiments are predicted by the established model. The prediction accuracy of the model for the kth batch of the experiment can be obtained by calculating the RMS [30] $S_{k}$, as shown below.

(22) $$S_{k}=\sqrt{\frac{\sum_{j=1}^{n}{\left(\hat{y_{j}}-y_{j}\right)}^{2}}{n-1}},$$

where k=1,…, 37 represents the batch of the predicted experimental data. The mean [12] of $S_{k}$ represents the prediction accuracy of the thermal error model. The calculations are as follows.

(23) $$S_{M}=\frac{1}{K}\overset{K}{\underset{k=1}{\sum }}S_{k},$$

where K=37.

**Figure 7:**
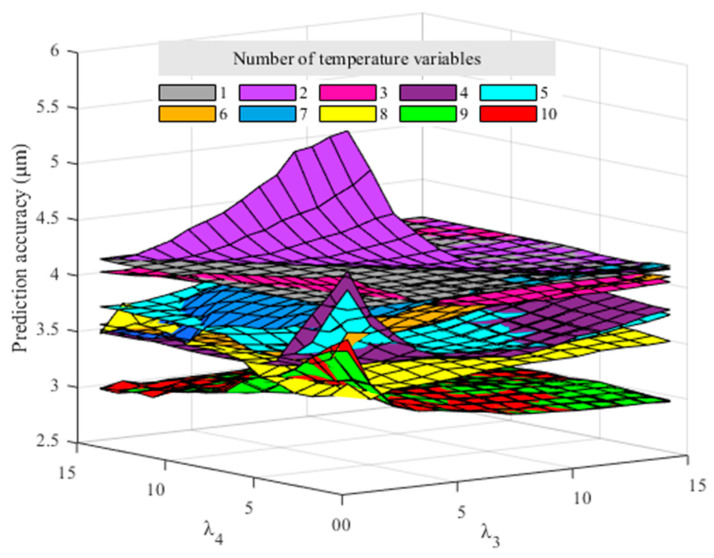
Prediction accuracy with different numbers of temperature variables and regularization coefficients.

The prediction accuracy of the established models with different numbers of MTVs and regularization coefficients can be calculated. To visualize the influence of the number of MTVs and the regularization coefficients, the prediction accuracy calculation results are drawn as a curved surface, as shown in Figure 7.

From Figure 7, it can be observed that the prediction accuracies are better (the smaller value means better accuracy) with a larger number of temperature variables. According to the calculation, when all 10 temperature variables are involved in the modeling, and the regularization coefficients $\lambda_{3}=15$ and $\lambda_{4}=14$, the prediction effect is the best. The best prediction accuracy and robustness are 2.93 μm and 1.52 μm, respectively. The LAENR algorithm can automatically eliminate unimportant or duplicate ones from the 10 temperature variables. The modeling results will be given later in Section 6.1 to show which unimportant temperature variables are eliminated. As a result, no separate TSP algorithm is needed for thermal error modeling, which would greatly simplify the thermal error modeling process.

### 5.3. Prediction Effects with Nine Batches of Experiments

Based on our preliminary results, we found that randomly selecting multiple batches of data for modeling cannot effectively improve the sufficiency of modeling data because some experimental data contain repetitive information. Therefore, in this paper, we select modeling data according to experimental conditions, that is, select the data of different experimental conditions for modeling. According to the analysis in Section 2.3, there are nine different experimental conditions, as shown in Table 3. Therefore, nine batches of experiments can be selected for modeling from each experimental condition. To verify the effectiveness of this approach, one batch of data is randomly selected from each experimental condition in Table 3. Thus, a total of nine batches are selected to build a thermal error model using the LAENR algorithm. The remaining data are predicted by the established model. The standard deviation [12] of $S_{k}$ is used to represent the robustness of the model. The calculations are as follows.

(24) $$S_{D}=\sqrt{\frac{\sum_{k=1}^{K}{\left(S_{k}-S_{M}\right)}^{2}}{K-1}},$$

where K=37 represents the total batch of the predicted experiments.

Then the prediction accuracy and robustness are calculated using Equations (22)–(24). The above analysis process is repeated 10 times to improve the credibility of the calculation results. The calculation results are shown in Table 5.

From Table 5, the average prediction accuracy and robustness are 2.82 μm and 1.74 μm, respectively. However, if we randomly select nine batches without considering the experimental conditions, the average prediction accuracy and robustness are 4.43 μm and 2.26 μm, respectively, based on 10 repetitions. This result proves that the method of selecting modeling data according to experimental conditions can achieve better prediction effects.

## 6. Prediction Effects Analysis

In this section, the modeling and prediction effects of the LAENR, ENR, ARX, LSTM, and GPR modeling methods are provided in Section 6.1 and Section 6.2, respectively. Then compensation experiments are carried out to show the effectiveness of the proposed modeling method in Section 6.3.

### 6.1. Modeling Effects of the Five Methods

**Figure 8:**
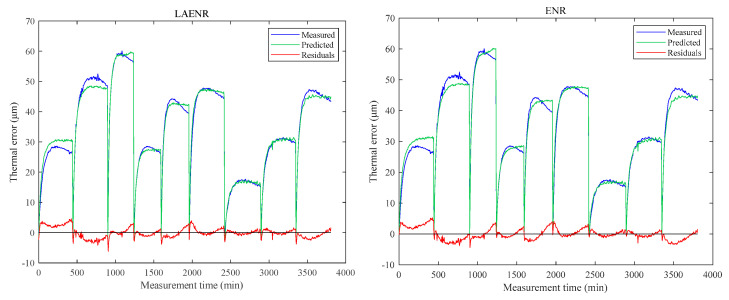

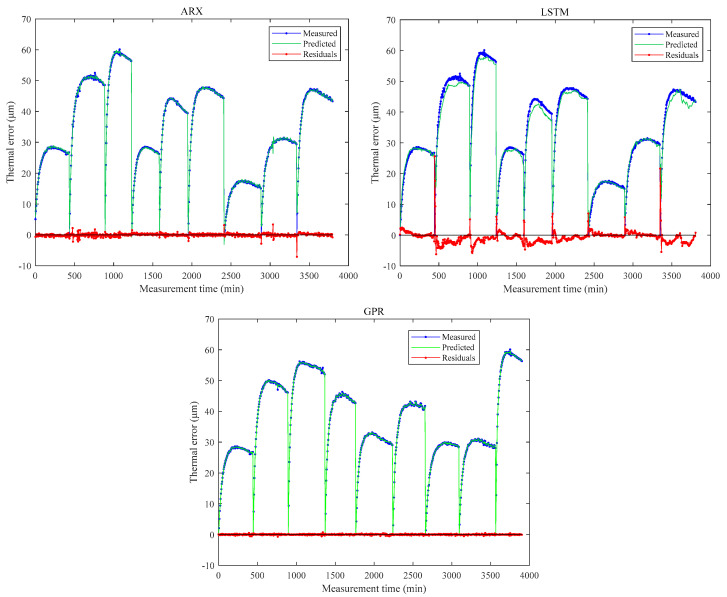
Fitting results of the five modeling methods.

In this subsection, the modeling result of the proposed LAENR is compared with that of the ENR, ARX, LSTM, and GPR methods. As mentioned in Section 5.2, the thermal error LAENR model is built using corresponding optimal regularization coefficients. In the same way, the optimal regularization coefficients of the ENR algorithm can be obtained as $\lambda_{1}=10$ and $\lambda_{2}=8$. Then all 10 temperature variables and the optimal regularization coefficients are applied to thermal error ENR model establishment. In addition, the thermal error ARX, LSTM, and GPR models are established according to reference [10]. The fitting curves of the thermal errors in the Z direction of the LAENR, ENR, ARX, LSTM, and GPR modeling methods are shown in Figure 8.

Further calculations show that the fitting accuracy of the LAENR, ENR, ARX, LSTM, and GPR methods are 1.85 μm, 2.06 μm, 0.53 μm, 1.87 μm, and 0.18 μm, respectively. In addition, coefficients of the ENR and LAENR models are shown in Table 6. The temperature variables $T_{5}$ and $T_{10}$ are zero in the ENR algorithm and the coefficients of temperature variable $T_{4}$ is zero in the LAENR algorithm. This shows that the LAENR modeling method has a stronger variable selection ability, thereby improving the robustness of the model.

### 6.2. Prediction Effects of the Five Methods

With the randomly selected nine batches of experiments according to experimental conditions, the LAENR, ENR, ARX, LSTM, and GPR thermal error models can be established. The remaining 37 batches of data are predicted. Then the prediction effects of these models can be calculated by Equations (22)–(24). To improve the reliability of the calculation results, the process of “select-predict-calculate” is repeated 10 times. The average values of these 10 calculation results are obtained, as shown in Table 7.

Table 7 shows that the values of $S_{M}$ and $\;S_{D}$ of the LAENR method are the smallest, in most cases, and are 3.66 μm and 1.52 μm in the X direction, 3.88 μm and 1.50 μm in the Y direction, 2.82 μm and 1.38 μm in the Z direction. These results prove that the proposed modeling method has the best prediction results. In particular, the prediction effects of the LAENR algorithm are better than those of the ENR algorithm, which means that the least absolute regression modeling method in this study can effectively improve the prediction ability. In addition, the LAENR method performs better than the deep-learning-based LSTM method in all directions. This shows our proposed method has the advantages of simple structure and better prediction performance. Finally, the prediction effects of the GPR algorithm are comparable to the proposed modeling method. However, the GPR model is complex with the iterative TSP selection procedure and lacks interpretability. It is also noteworthy that while the GPR and ARX methods have better modeling accuracy than the LAENR method as shown in Section 6.1, they do not perform better than the proposed method in prediction accuracy. One possible reason is that both GPR and ARX methods overfit the data and their prediction accuracies decrease with unseen test data.

### 6.3. Compensation Verification Experiments

The established LAENR thermal error prediction model was transmitted to the CNC system of the research object. Then three batches of thermal error compensation verification experiments for air cutting are carried out and recorded as V1–V3. The spindle of V1–V3 idles at speeds of 2000, 4000, and 6000 rpm, respectively. The feed rate of the three experiments is 1500 mm/min. In each batch of the experiment, the temperatures of the machining center are measured in real-time and transferred to the prediction model. Then the thermal error can be predicted and compensated in real time.

**Figure 9:**
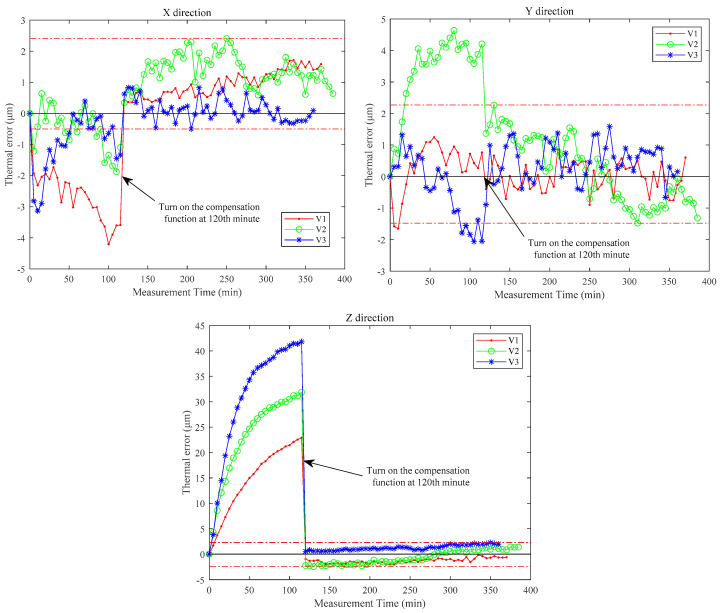
Thermal error measurement results after compensation.

The thermal error compensation function is turned on after the machine has been running for 2 h to visually show the actual compensation effect. The thermal error changes of V1–V3 are plotted in Figure 9. After compensation, the ranges of thermal errors in the X, Y, and Z directions are −0.50–2.41 μm, −1.48–2.27 μm, and −2.45–2.26 μm. The effectiveness of the proposed modeling method is proved.

## 7. Conclusions

To address the growing complexity and lack of interpretability of thermal error modeling, this study proposes an effective and practical method based on regularized regression. The optimal number of MTVs and regularization coefficients are analyzed based on experimental data. The prediction models are established by experimental data under different experimental conditions. The proposed modeling method is compared with those of the ENR, ARX, LSTM, and GPR algorithms. The calculation results show that the proposed modeling method achieves the best prediction accuracy and robustness in all X, Y, and Z directions. Finally, the effectiveness of the proposed modeling method in real-world applications is proved by compensation experiments, which can control the thermal errors with ±3 μm.

For future research, the modeling method with other normalizations of temperature differences between experiments will be studied first. Second, in our verification experiments, the error drifted out of the tolerance bandwidth after a certain amount of time. To address this issue, the adaptability of the thermal error prediction model by online updating will be considered as an important future work to improve the ability to maintain prediction accuracy. Last, a universal modeling method applicable to most types of machine tools will be studied.

## Figures and Tables

**Table 1:** The installation locations and functions of sensors.

Sensors	Installation Site	Function
T1–T5	Front bearing of spindle	Bearing temperature measurement
T7, T8	Spindle motor	Spindle motor temperature measurement
T6, T9	Spindle box	Spindle box temperature measurement
T10	Machine frame	Ambient temperature measurement

**Table 2:** Experimental parameters.

Batch	Initial Environment Temperature/°C	Environment Temperature Rise/°C	Spindle Speed/rpm	Feed Speed/mm·min^−1^
K1	3.7	2.2	4000	1500
K2	5.3	5.1	4000	
K3	6.2	3.8	6000	
K4	10.6	1.4	2000	
……				
K43	27.6	1.3	6000	
K44	28.5	1.1	4000	
K45	28.9	0.8	6000	
K46	32.2	1.5	2000	

**Table 3:** Nine different experimental conditions with the corresponding batches.

Number	Environmental Temperature	Spindle Speed (rpm)	Included Batches of Data
1	below 13.2 °C	2000	K4, K7, K10, K12, K14, K15
2	below 13.2 °C	4000	K1, K2, K5, K9, K11, K13
3	below 13.2 °C	6000	K3, K6, K8, K18
4	13.2–22.7 °C	2000	K17, K24, K25, K30
5	13.2–22.7 °C	4000	K21, K22, K26, K28, K29
6	13.2–22.7 °C	6000	K16, K19, K20, K23, K27
7	over 22.7 °C	2000	K31, K33, K39, K40, K41, K46
8	over 22.7 °C	4000	K32, K34, K38, K42, K44
9	over 22.7 °C	6000	K35, K36, K37, K43, K45

**Table 4:** Model results with different numbers of temperature variables used the selected nine batches of experiments.

Number of Temperature Variables	Temperature Variables	$\mathrm{Coefficients}\;\beta_{i}$	Fitting Accuracy (μm)
2	T1, T5	4.73, 9.45, −5.76	4.02
3	T1, T5, T2	5.18, 9.90, 0.00, −6.70	3.68
4	T1, T5, T2, T4	1.37, 9.25, 3.81, −3.44, −6.56	3.23
5	T1, T5, T2, T4, T3	1.67, 8.39, 3.67, −0.86, −4.99, −3.34	3.30
6	T1, T5, T2, T4, T3, T6	3.20, 6.75, 0.53, −1.57, −2.23, 2082, −4.21	3.08
7	T1, T5, T2, T4, T3, T6, T9	1.25, 5.19, 1.28, −1.69, −1.39, 0.37, −5.88, 1.32	2.88
8	T1, T5, T2, T4, T3, T6, T9, T8	0.00, 1.65, 2.36, −1.59, 2.94, 3.61, −5.82, −6.61, 5.67	2.23
9	T1, T5, T2, T4, T3, T6, T9, T8, T7	−2.33, 2.36, 5.80, −4.29, 0.00, 1.32, −1.67, −1.58, 4.94, −5.31	1.85
10	T1, T5, T2, T4, T3, T6, T9, T8, T7, T10	−2.20, 2.05, 6.08, −4.17, 0.00, 1.28, −1.75, −1.81, 5.08, −4.98, −0.34	1.85

**Table 5:** Prediction effects with nine batches of experiments (Unit: μm).

Number of Calculations	1st	2nd	3rd	4th	5th	6th	7th	8th	9th	Average
Prediction accuracy	2.94	2.80	2.84	2.61	2.99	2.71	2.96	2.70	2.85	2.82
Robustness	1.58	1.93	1.71	1.81	1.37	2.18	1.28	1.73	2.09	1.74

**Table 6:** Modeling coefficients of the ENR and LAENR methods.

Modeling Coefficients	$\beta_{0}$	$\beta_{1}$	$\beta_{2}$	$\beta_{3}$	$\beta_{4}$	$\beta_{5}$	$\beta_{6}$	$\beta_{7}$	$\beta_{8}$	$\beta_{9}$	$\beta_{10}$
ENR	−1.84	2.85	3.28	−1.44	0.35	0.00	−1.74	−0.68	4.24	−5.32	0.00
LAENR	−2.19	2.06	6.06	−4.17	0.00	1.29	−1.75	−1.82	5.08	−4.97	−0.34

**Table 7:** Prediction effects of the five compared methods (Unit: μm).

Model	Direction					
	X (μm)		Y (μm)		Z (μm)	
	$S_{M}$	$S_{D}$	$S_{M}$	$S_{D}$	$S_{M}$	$S_{D}$
LAENR	3.66	1.52	3.88	1.50	2.82	1.38
ENR	3.89	1.90	4.01	1.72	3.03	1.61
ARX	5.19	2.11	5.43	2.22	4.55	2.08
LSTM	3.93	1.74	4.01	1.88	3.47	1.60
GPR	3.78	1.43	3.99	1.87	2.88	1.32

## Data Availability

Not applicable.

## Abbreviations

TSP	Temperature-Sensitive Point
MTVs	Modeling Temperature Variables
GPR	Gaussian Process Regression
NN	Neural Network
LSTM	Long Short-Term Memory
LASSO	Least Absolute Shrinkage and Selection Operator
ENR	Elastic-Net Regression
LAENR	Least Absolute Elastic-Net Regression
RMS	Root Mean Square
